# Nanocoatings for Chronic Wound Repair—Modulation of Microbial Colonization and Biofilm Formation

**DOI:** 10.3390/ijms19041179

**Published:** 2018-04-12

**Authors:** Mara Mădălina Mihai, Mădălina Preda, Iulia Lungu, Monica Cartelle Gestal, Mircea Ioan Popa, Alina Maria Holban

**Affiliations:** 1 Department of Oncologic Dermatology and Allergology, “Carol Davila” University of Medicine and Pharmacy, 030167 Bucharest, Romania; drmaramihai@gmail.com; 2 Department of Dermatology, “Elias” University Emergency Hospital, 011461 Bucharest, Romania; 3 Department of Microbiology, Faculty of Medicine, “Carol Davila” University of Medicine and Pharmacy, 030167 Bucharest, Romania; madalina.prd@gmail.com (M.P.); mircea.ioan.popa@gmail.com (M.I.P.); 4 Cantacuzino National Medico-Military Research and Development Institute, 050096 Bucharest, Romania; 5 Department of Biomaterials and Medical Devices, Faculty of Medical Engineering, University Politehnica of Bucharest, 060042 București, Romania; iulia.lunguu@gmail.com; 6 Department of Infectious Diseases, College of Veterinary Medicine, University of Georgia, Athens, Georgia, GA 30602, USA; mcarges@gmail.com; 7 Department of Microbiology, Faculty of Biology, University of Bucharest, 030018 București, Romania; 8 Research Institute of the University of Bucharest (ICUB), 050107 Bucharest, Romania

**Keywords:** chronic wound, biofilm formation, tolerance, antimicrobial nanoparticles, nanocoatings

## Abstract

Wound healing involves a complex interaction between immunity and other natural host processes, and to succeed it requires a well-defined cascade of events. Chronic wound infections can be mono- or polymicrobial but their major characteristic is their ability to develop a biofilm. A biofilm reduces the effectiveness of treatment and increases resistance. A biofilm is an ecosystem on its own, enabling the bacteria and the host to establish different social interactions, such as competition or cooperation. With an increasing incidence of chronic wounds and, implicitly, of chronic biofilm infections, there is a need for alternative therapeutic agents. Nanotechnology shows promising openings, either by the intrinsic antimicrobial properties of nanoparticles or their function as drug carriers. Nanoparticles and nanostructured coatings can be active at low concentrations toward a large variety of infectious agents; thus, they are unlikely to elicit emergence of resistance. Nanoparticles might contribute to the modulation of microbial colonization and biofilm formation in wounds. This comprehensive review comprises the pathogenesis of chronic wounds, the role of chronic wound colonization and infection in the healing process, the conventional and alternative topical therapeutic approaches designed to combat infection and stimulate healing, as well as revolutionizing therapies such as nanotechnology-based wound healing approaches.

## 1. Introduction

Chronic wounds, such as venous or arterial ulcers, diabetic foot ulcers, pressure sores, and non-healing surgical wounds, have a significant impact on a patient’s quality of life, and are an important economic burden. Chronic wounds are associated with chronic mono- or polymicrobial biofilm infections and are characterized by tolerance and resistance to antimicrobials. Within biofilms, microbial species can establish relationships of cooperation and competition; finally evolving into an elaborate and functional adapted communities. Their interaction with the host’s immune system or with therapeutic agents contributes to the complexity of the wound ecosystem and modulates the healing potential.

Wound debridement and the topical application of antibiotics or other antimicrobial substances are the conventional methods usually considered to eradicate wound infection. The main disadvantage of recurrent antibiotic use in the context of delayed wound healing and frequent hospitalizations is exacerbated by the rising risk of therapeutic resistance. Alternative treatments to be considered include immune-based antimicrobial molecules (polypeptides like defensins), the use of microorganisms (probiotics and bacteriophages), phototherapy (blue light, ultraviolet light, and others), and photodynamic therapy (light exposure after locally applied photosensitizing dyes) [1]. 

Based on current knowledge, the ideal therapeutic agent should achieve multiple objectives, including antimicrobial, immunomodulatory, and regenerative effects [2]. Nanotechnology reveals promising openings, either by the intrinsic antimicrobial properties of nanoparticles or by their function as drug carriers. However, nanotechnology is also important for the development of nanostructured bioactive dressings and coatings [2]. Nanoparticles are active at low concentrations toward a large variety of infectious agents. Importantly, they are unlikely to provoke the emergence of resistance and have the ability to modulate microbial colonization and biofilm formation [2].

In this review, we compile the latest literature starting with wound pathogenesis, with special emphasis on biofilms, and describe both the conventional approaches and new approaches in wound healing, emphasizing the novel nano-technological approaches that are not only cutting edge but also are promising in the field. We finalize with our perspectives and remarks.

## 2. Pathogenesis of Chronic Wounds

### 2.1. Pathophysiology of Acute Wound Repair

The human skin acts as a protective barrier against aggressive external factors, playing a pivotal role in the maintenance of various metabolic processes, such as thermoregulation, body fluid homeostasis, immune control, and others [3]. Injuries caused by external (chemical, physical, mechanical) and/or internal (vascular deficiency) factors can disrupt the continuity of the cutaneous barrier, creating vascular supply destruction and an available source of nutrients for microbes [3]. Severe trauma might cause extensive wounds with a higher susceptibility toward systemic immunosuppression, wound infection, sepsis, and even death. The source of microbial colonization is not only exogenous, from the environment, with nosocomial and community-acquired strains but can also be caused by normal microbiota of the individual, either cutaneous or mucosal, after a disruption in their normal balance.

**Figure 1:**
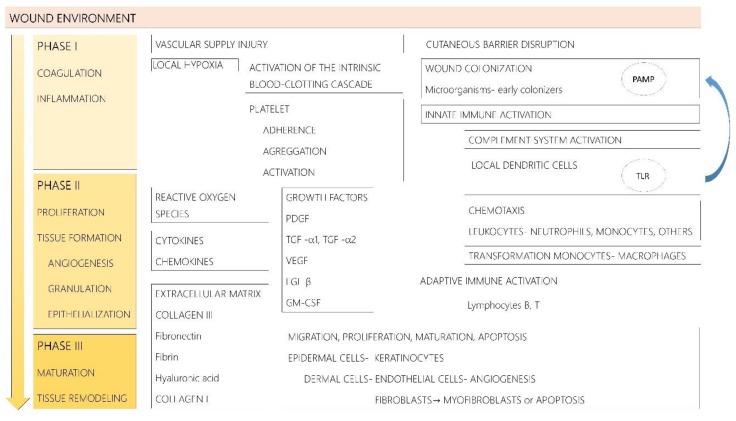
Acute wound healing consists of the coagulation and inflammatory phase, the proliferation and tissue formation phase, and the maturation and remodeling phase. Abbreviations: PDGF—platelet-derived growth factor; TGF-α1, TGF-α2—transforming growth factors alpha 1 and alpha 2; VEGF—vascular endothelial growth factor; FGF-β—fibroblast growth factor beta; GM-CSF—granulocyte-macrophage colony-stimulating factor; TLR—Toll-like receptor; PAMP—pathogen-associated molecular pattern.

Healing is a complex process that involves an orderly and timely sequence of events, divided into three differential phases: the coagulation and inflammatory phase, the proliferation and tissue formation phase, and the maturation and remodeling phase (Figure 1). The phases are not separated but rather they overlap and influence each other. The healing phases are strictly followed by acute wounds, which progress through each stage towards successful epithelialization and wound closure [4].

The coagulation/inflammatory phase starts straight after the injury in an attempt to minimize the damage that occurred. It involves platelet adherence to the damaged vessels and aggregation, blood clotting, and immune activation. Thrombocytes will immediately start to produce different cytokines, chemokines, and growth factors [5]. Among the released growth factors, platelet-derived growth factor (PDGF) and transforming growth factors alpha 1 and alpha 2 (TGF-α1, TGF-α2) are involved in attracting inflammatory cells [5]. In the early steps, local dendritic cells express pattern recognition receptors, such as Toll-like receptors (TLRs) that recognize the pathogen-associated molecular patterns (PAMPs) of colonizing microbes. This interaction leads towards innate immune activation, characterized by a significant recruitment of neutrophils and monocytes to the site of damage [6]. The reactive oxygen species released by the attracted leukocytes are essential for the antimicrobial action at the wound site [5]. The complement system is also activated [7]. The initial changes are followed by the transformation of monocytes into macrophages and the migration of fibroblasts, leukocytes, keratinocytes, and endothelial cells, as well as by the local accumulation of growth factors, to the wound [7,8]. Later on, there is a change towards adaptive immunity involving lymphocytes (T-cells and B-cells) and a complex cascade of cytokines and chemokines [7]. This leads to the second phase of proliferation and tissue formation, which includes three important steps: re-epithelialization, angiogenesis, and development of granulation tissue. Local cytokines and growth factors induce re-epithelialization by the activation of fibroblasts, keratinocytes, epithelial cells, and stem cells. Moreover, fibroblasts produce collagen and other molecules that form a new extracellular matrix, an appropriate scaffold for cell adhesion, growth, and differentiation [5,7]. The extracellular matrix is composed of fibronectin, fibrin, hyaluronic acid, and collagen type III at the beginning replaced later with collagen type I [5]. To support all the cells involved in the healing process, the blood supply plays a key role; this is why angiogenesis starts immediately after a blood vessel injury occurs [5]. During angiogenesis, multiple endothelial growth factors such as PDGF, vascular endothelial growth factor (VEGF), fibroblast growth factor beta (FGF-β), and granulocyte-macrophage colony-stimulating factor (GM-CSF) act on the development of new blood vessels, with an increase in nutrient and oxygen availability to the developing tissue [7]. This is a very complex process involving, among others, the degradation of the basement membrane of the blood vessels, the migration of the endothelial cells to the wound site, and the formation of new blood vessels, all this under the influence of different substances, factors, and even cell progenitors, such as the endothelial progenitor cells [9]. At the end of this phase, the fibroblasts differentiate into myofibroblasts or undergo apoptosis [5,7,10].

Finally, tissue maturation and remodeling occur with a change in composition of primarily type I collagen that produces the mature scar [3,4]. In this phase, cellular apoptosis plays an essential role [11]. If the apoptotic process is malfunctioning, several wound healing pathologies can develop, such as hypertrophic scars or keloids [5].

It is important to mention that the cutaneous injury can lead to neuroendocrine changes with the secretion of stress mediators, such as cortisol, epinephrine, norepinephrine, acetylcholine, catestatin, substance P, and α-melanotropin [6]. It was stated that these molecules alter the cutaneous microbiota with a prevalence of harming bacteria contributing to wound infection and impaired healing [6].

### 2.2. Pathophysiology of Chronic Wound Repair

Chronic wounds, such as venous or arterial ulcers, diabetic foot ulcers, pressure sores, and non-healing surgical wounds, are defined by the lack of improvement within four weeks and the lack of restoring a functional result after a period of three months [10]. The incidence within the general population is between 1% and 4% [4]. In the elderly population, chronic wounds increase the comorbidity and mortality.

Failure to rebuild the skin integrity may be due to a large number of potential contributory causes. Local factors that affect tissue oxygenation or inflammation—vascular disease (arterial or venous chronic pathologies, phlebitis, thrombosis, vasculitis), immobility, severe trauma or repeated injuries in the same region, chronic pressure, chronic edema, colonization by opportunistic pathogens, chronic infection with the development of biofilms, and others—can increase the risk of healing failure [6,12]. However, systemic factors that affect the overall health of the individual can play an important role in the healing process (for example, diabetes, diet and obesity, hypertension, chronic inflammatory diseases, and others) [6,12]. 

Depending on the type of wound and the host factors, the pathophysiological process is different. Venous ulcers are the result, amongst others, of valvular incompetence, which leads to an increase in the blood pressure and vessel permeability, consequently producing an accumulation of fibrin and a decrease in collagen synthesis [5]. Arterial ulcers are produced by an insufficient blood flow [5]. Hospitalization and long-term decubitus are particularly involved in the development of pressure ulcers [4]. Pressure ulcers are the result of pressure applied to the skin and underlying tissue, being more frequent in patients with decreased mobility and sensory perception [5]. The common characteristic of all of these wounds is chronic inflammation, most frequently triggered by the presence of bacteria. During the process, inflammatory cells produce reactive oxygen species, which will lead to the damage of the extracellular matrix [5].

Chronic wounds fail to heal in the physiologically ordered sequence of events, most of them entering a vicious cycle of inflammation and infection [10]. Non-healing wounds display important morphological, clinical, biochemical, and microbiological differences when compared with both acute and healing wounds [3]. The tissue damage and repair are misbalanced, resulting in the ulcer’s lodging in a particular wound stage, most frequently in the inflammation or granulation stages, and consequently impairing progression toward wound healing [5]. Studies have shown that cells from chronic wounds have lower multiplying rates and are similar to senescent ones [10,13]. Moreover, the fibroblasts from chronic wounds are less responsive to PDGF and transforming growth factor beta (TGF-β) [10,13]. Another characteristic of chronic wounds is the reduced angiogenesis and, hence, a low blood supply that results in hypoxia [10,13]. Other factors involved in the elongation of the healing process are an increased concentration of iron that results from the destroyed erythrocytes and mitochondrial dysfunction [10,13]. It was stated that a hallmark of chronic wounds is represented by the insufficient bioavailability of growth factors, critically important for normal injury repair; the deficiency is probably caused by diminished synthesis and/or excessive degradation [2,13]. This observation might suggest the potential use of exogenous growth factors in the treatment of non-healing wounds [2,13].

### 2.3. Microbial Colonization and Wound Healing

One significant factor that influences the healing process of wounds is characterized by the microbial colonization of the wound. Although initially the host’s immune system controls the proliferation of bacteria, further on the microorganisms develop biofilms and a tolerance toward immune defense mechanisms. Therefore, a microbial infection may occur, which could lead to impaired wound healing [14].

The exact role of bacteria in delayed wound healing remains controversial because there is a discrepancy in the published scientific results. It was previously believed that high microbial levels are correlated with the inability to heal. It was stated that wound infection risk is proportional to the number of colonizing microbes (N) multiplied by their virulence (V) and divided by host immunity (I): infection risk = (N × V)/I [14]. Several factors could influence each of these variables: environmental factors such as temperature and humidity; wound characteristics such as depth, vascularization; patient compliance to treatment by applying proper care and cleaning of the wound. On the other hand, recent research has shown that an association between bacteria and delayed healing does not always exist, and that bacteria might be responsible in some situations of a positive wound outcome, for example, after the topical contact with bacterial lipopolysaccharides [15,16,17,18]. It was suggested that the qualitative characteristics of the microbial ecosystem can modulate its impact on wound healing [18].

What if there were no bacteria? In their study of acute wound healing, Canesso et al. (2014) observed that the total absence of commensal microbiota in germfree mice positively affects the cutaneous wound healing process, being significantly accelerated and scarless [19]. The results might be explained partially because of the reduced accumulation of neutrophils, increased accumulation of alternatively activated healing macrophages, and better angiogenesis at the wound site [19].

The microorganisms most frequently isolated from chronic wounds by traditional culturing techniques are represented by *Staphylococcus aureus* (*S. aureus*) and *Pseudomonas aeruginosa* (*P. aeruginosa*), followed by various species of *Enterobacteriaceae* such as *Escherichia coli*, *Klebsiella* spp., *Proteus* spp., *Enterobacter* spp., *Morganella morganii*, *Citrobacter freundii*, *Serratia marcescens* and *Providencia* spp., *Enterococcus* spp., *Streptococcus* spp., and rarely *Corynebacterium* spp. or *Acinetobacter baumannii* [20,21,22].

Molecular techniques, such as 16S DNA sequencing, revealed significant differences compared to the traditional method, with the identification of up to ten times more unique genera (145/14) in the following order: *Staphylococcus*, *Corynebacterium*, *Pseudomonas*, *Clostridium*, *Bacillus*, *Enterococcus*, and others [23].

By aerobic culturing tests, it was revealed that in over 50% of chronic wound samples a single bacterial species was present, while the association of two bacterial species was observed in over 20% of cases [22,23]. The most frequent bacterial association was between *P. aeruginosa* and *S. aureus* [22,23].

Regardless of bacterial counts, specific strains have been related to chronic wound infections and delayed healing (e.g., *Staphylococcus*, *Streptococcus*, and *Pseudomonas*), a fact that might be explained by the expression of potent virulence factors that act to destroy the tissue [24]. As an example, Shettigar et al. (2016) observed that *S. aureus* isolates from monomicrobial and polymicrobial infections of diabetic foot ulcers differed significantly in the expression of their virulence potential [25]. Moreover, preclinical models in small and large animals revealed that individual biofilms by *S. aureus*, *S. epidermidis*, and *P. aeruginosa* delay wound re-epithelialization, which is directly attributed to the biofilm formation [26,27].

It was previously observed that co-infection with *P. aeruginosa* and *S. aureus* results in delayed wound healing compared to single species contaminations [28,29]. Pastar et al. (2013) proved that expression of the *pvl* gene, coding the staphylococcal Panton–Valentine leukocidin, a virulence factor that causes leukocyte destruction and tissue necrosis, was significantly upregulated in the co-infection of *S. aureus* with *P. aeruginosa* [28]. Hotterbeekx et al. (2017) described the co-pathogenicity of *S. aureus* with *P. aeruginosa* as “a network of evasion, counter-inhibition, and subjugation” [30]. Although the two species might initially develop within a complex relationship of competition for space and nutrients, after longer periods of co-existence, as in the case of chronic wound infections, the two bacterial phenotypes might become better adapted to the presence of each other and develop synergistic effects [25].

Bertesteanu et al. (2014) emphasized the complex synergistic or antagonistic interactions within polymicrobial wound infections, with an impact over several biological processes such as microbial adherence, resource utilization, intercellular communication by quorum-sensing-mediated cross-talking, the expression of different virulence phenotypes, and host immunomodulation [2]. These complex interactions are clearly illustrated by the concept of microbial biofilms discussed below.

### 2.4. Bacterial Biofilms and Wound Healing

Biofilms are defined as tridimensional microbial consortia where microorganisms are closely attached to a surface or to one another within an exopolysaccharide matrix (EPS) [14,31,32]. Biofilms are “structurally and dynamically complex biological systems” [33] and might be imagined as primitive, multicellular organisms characterized by defense, adaptation, and pathogenesis [17,34,35]. It was stated that biofilms impact chronic wound healing by delaying the inflammatory and maturation phases [17,31]. Moreover, polymicrobial biofilms impair wound healing more significantly than monomicrobial biofilms, potentially due to synergistic interactions among bacteria expressing different virulence phenotypes [36]. An interesting hypothesis suggested that biofilms might also be commensal and able to maintain wound homeostasis [5].

Biofilm development consists of five main steps. The first step is represented by wound contamination with the adhesion of microorganisms and the colonization of host tissues. In the beginning, the adherence is reversible. The microorganisms can easily detach, either spontaneously or by physical or chemical methods, and are susceptible to antibiotics. Further on, a mucopolysaccharide matrix composed of microbial and host molecules, such as polysaccharides, lipids, proteins, and nucleic acids, will lead to a mature biofilm that is characterized by tolerance to antibiotics and immune control [37]. Within a biofilm, inter-bacterial communication is accomplished via complex signaling networks, named quorum sensing, consisting of small organic compounds in response to population density [37,38]. The architecture of the biofilm can be different depending on the bacteria involved [39].

Wound inflammation appears as a consequence of delayed healing and microbial colonization. Host response via innate and adaptive immunity plays an important role in the establishment and course of the biofilms [40]. It was stated that microbial biofilms are partially but not entirely protected against the action of neutrophils, including phagocytosis, degranulation, and the development of neutrophil extracellular traps [41]. However, the efficiency of the immune defense is dependent on several factors such as microbial virulence, microbial composition and diversity, and composition of the extracellular matrix [41]. Immune pressure can contribute to the selection of more resistant or more virulent strains and, therefore, contribute to the physical and ecological stability of biofilms [41]. Likewise, neutrophil enzymes can degrade collagen and other proteins and, as a result, cause harm to the host tissues and delay wound healing [41].

Mature biofilms constantly release planktonic cells that migrate and colonize surrounding areas to establish novel biofilms [32]. Frequently, patients with chronic wound infections develop acute episodes with clinical signs and symptoms of infection: local erythema, edema, pain, wound malodor, and purulent secretions. In rare cases, patients might develop signs of systemic infection and septicemia: fever, confusion, tachypnea, and eventually death if not treated [14].

Biofilms have been identified in many animal and human infections, such as caries [42], cystic fibrosis [40], and otitis media [43]. In 2012, it was reported that microbial biofilms are present in at least 80% of surgical site infections [44]. In 2008, James et al. published that 60% of the chronic wounds sampled in their study contained a biofilm, indicating the great prevalence of this type of infection in chronic wounds [45]. The Centers for Disease Control and Prevention estimate that 80% of all human infections are associated with a biofilm and at least 65% of them are of nosocomial origin [46].

Several research groups evaluated the ability of drug-resistant microorganisms isolated from chronic wounds to produce biofilms. Using the clinical BioFilm Ring Test [47], Di Domenico et al. (2017) observed that *S. aureus* and *P. aeruginosa* showed a comparable ability to develop biofilms, with a moderate or high intensity in 94.6% and 73.7% of strains, respectively. This is significantly superior to *Enterobacteriaceae* species such as *K. pneumoniae* (60.0%), *A. baumannii* (58.3%), and *E. coli* (53.3%) that were mainly weak biofilm producers [48]. Interestingly, while some of the strains did not produce biofilms in vitro, they were associated in vivo as a moderate or high biofilm-producing strain [48], reinforcing the need to focus research on polymicrobial colonization as a whole and its impact on wound healing.

One of the most important characteristics of biofilms is represented by their tolerance to environmental stressors, including antibiotic therapy. While resistance to antibiotics can be genetically acquired or due to epigenetic changes; tolerance can revert to susceptibility after a phenotypic change from biofilm-forming bacteria, toward planktonic (free-floating) bacteria [9]. Bacterial tolerance is determined by at least five factors, either independent or combined: the reduced antibiotic diffusion through the biofilm matrix; the enzymatic breakdown of antibiotics within biofilms; the emergence of phenotypic bacterial variants with low metabolic rates called “persisters”, which are also tolerant to antimicrobial compounds acting on bacterial division; the activation of bacterial stress-response genes leading to increased tolerance towards environmental stresses; the occurrence of genetic changes due to the close proximity of bacterial species, hypermutability, and an increased horizontal gene transfer, encouraging tolerance to environmental stress and even the emergence at higher rates of resistant bacterial strains [49,50].

## 3. Limitations of Current Wound Therapy

An effective wound management strategy should aim to prevent and treat infections, as well as to promote healing and prevent scarring [7]. During the inflammatory and remodeling phases, an adequate balance between tissue damage and tissue repair should be maintained. There are a number of available therapeutic options (Table 1).

Most infections can be prevented with the correct prophylaxis; however, high antibiotic pressure has selected for highly resistant bacteria. The major risk of chronic wounds is related to nosocomial infections and high antibiotic-resistant pathogens [4].

Although it represents the first line of treatment in acute infections, antibiotic therapy administered systemically or topically in non-toxic doses cannot eradicate biofilm infections and there is no evidence to support its further use. Biofilm antibiotic susceptibility testing revealed that due to microbial tolerance, bactericidal concentrations reached values 1000 times higher compared to the ones for planktonic bacteria [46,47]. Moreover, it was proven that systemic antibiotherapy has a lower availability at the site of infection and can increase the risk of microbial drug resistance [7].

There is a fine line between the beneficial effects of antibiotic therapy, such as the eradication of planktonic bacteria and biofilm disruption, and its pathogenic effects, such as the development of bacterial resistance and tolerance to antibiotics. There are several experimental studies that brought proof on the fact that fluoroquinolones, tetracycline, rifampin, daptomycin, and vancomycin can rapidly diffuse into the deeper levels of the biofilms and contribute to their disruption [48], but these results are achieved only with other therapeutic approaches (topical antiseptics, wound debridement, and others).

A thorough wound debridement, either mechanical, enzymatic, or biological, represents an important stage of the recommended therapeutic approach in the case of chronic biofilm infection of non-healing venous ulcers, pressure ulcers, diabetic ulcers, and burns [51]. Debridement is the most efficient option for reducing wound bioburden and it was shown to favor the healing process [51]. Sharp wound debridement might also “open a time-dependent window”: it physically disrupts the mucopolysaccharide matrix, it destabilizes the biofilm’s architecture, it promotes bacterial detachment, and it increases antimicrobial delivery and susceptibility [52]. Nevertheless, wound debridement is not recommended as single anti-biofilm therapy because it might cause additional wound trauma, promote the inoculation of infection into deeper tissues, and have only a temporary positive effect [53]. Moreover, debridement should not be considered in arterial and surgical chronic wounds [50].

Recent guidelines on wound care emphasize the importance of topical antiseptic therapy and personalized dressings. Although antiseptics such as povidone-iodine, chlorhexidine, hydrogen peroxide, boric acid, silver sulfadiazine or nitrate, sodium hypochlorite, and mafenide acetate are intrinsically cytotoxic to bacteria, fungi, and other microorganisms, they might also damage host cells and adversely affect wound healing [7]. Octenidine dihydrochloride and polyhexamethylene biguanide (polyhexanide) seem to be the most efficient and well-tolerated antiseptics [54], while the latter is considered the best promoter of wound healing [55].

With the increasing incidence of chronic wounds and, implicitly, of chronic biofilm infections, there is a need for alternative therapeutic agents. One such therapy is represented by bacteriophage therapy, viruses with a specific tropism for bacterial cells, with bactericidal effects. This therapy may be efficient against polymicrobial biofilm-mediated infections based on its high host specificity [2]; however, the main disadvantage refers to the biologically-associated risks, production costs, and the fact that such formulation could be applied in low amounts as a topical treatment.

Other alternative antimicrobial therapies are antimicrobial peptides (defensins, magainins, cecropins) or natural products that possess antimicrobial properties (essential oils, honey, and others) [2]. Despite their antimicrobial efficiency, they could be utilized only in low amounts in humans and animals and most of them have low stability or increased volatility, thus requesting delivery shuttles and stabilizing agents [56].

Polymicrobial vaccines have not proven to be as efficient as expected in clinical trials. Bertesteanu et al. (2014) proposed an optimization of the vaccines: to target shared microbial determinants and to simultaneously attenuate the potential virulence of different co-infecting species in order to reduce the risk of recurrence [2].

A recent meta-analysis of animal studies showed that probiotic therapy accelerates wound healing [57]. As an example, *Lactobacillus plantarum* prevents wound colonization, biofilm development, and interferes with the quorum sensing of *P. aeruginosa* [58]; it also is of comparable efficacy with silver sulfadiazine treatment [59]. Moreover, *L. plantarum* enhances phagocyte activity and tissue repair [58]. However, there is insufficient data to make a clear recommendation on the use of probiotics in clinical practice.

Antimicrobial light and ultrasound-based wound therapy, phototherapy, shockwave, and stem cell-based therapy represent other viable options that are rarely applied in clinical practice [2].

Based on current knowledge, an ideal agent should achieve antimicrobial, immunomodulatory, and regenerative effects [2], aiming to disrupt the pathogenic mechanisms and to enhance a beneficial environment for healing. It should achieve an optimal delivery to the target [9], either within biofilms or within host tissues, and increase the patient’s quality of life [45]. In regard to the antimicrobial action, an ideal therapeutic approach should accomplish the following: achieve targeted antimicrobial activity without suppressing the beneficial bacteria and cutaneous normal microbiota; reduce the bacterial load below the critical colonization limit and, therefore, prevent wound infection [45]; modulate microbial colonization, attachment, and biofilm development; modulate and promote beneficial bacterial phenotypes; modulate the in vivo expression of virulence factors by the bacterial consortia (different from the intrinsic virulence of each single microbial species in a planktonic growth state) [2]; and modulate interbacterial and host-microbiome interactions. The agent should achieve immunomodulatory action by supporting the host’s defense mechanisms [45], as well as regenerative effects, by the enhancement of wound healing and tissue regeneration. Importantly, to achieve optimal functional results, adverse reactions such as allergy or intolerance should be rapidly diagnosed [9].

## 4. Advanced Wound Dressings and Coatings

Although in recent years numerous materials were developed to create better wound dressings, the ideal coating has not been created yet. Current trends in wound dressing design rely on some particular traits that should be fulfilled by an advanced dressing: (i) creates a moist, clean, warm environment; (ii) provides hydration to dry skin; (iii) removes excess exudates; (iv) prevents desiccation and is atraumatic; (v) allows for gaseous exchange; (vi) is impermeable to microorganisms; (vii) is free of toxic or irritant particles; (viii) can conform to wound shape; and (ix) is easy to use with minimal pain during application and removal [60].

Since microbial contamination represents one of the major risks for delayed wound healing, recent studies are investigating efficient methods to prevent microbial colonization and biofilm formation at the wound.

Significant research was made in the last decade to develop bioactive polymeric coatings and dressings for difficult-to-heal wounds.

**Figure 2:**
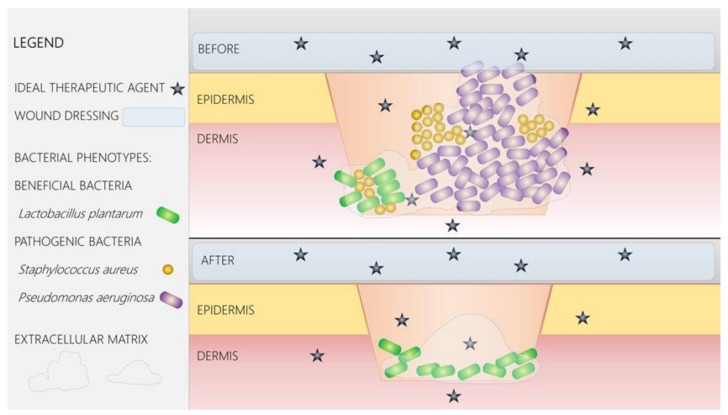
Nanoparticles embedded within bioactive wound dressings could enhance the delivery to the target of beneficial molecules with antimicrobial, immunomodulatory, and regenerative effects. In regard to the antimicrobial action, an ideal therapeutic agent should destroy pathogenic bacteria but also modulate microbial colonization, attachment, and biofilm development; modulate and promote beneficial bacterial phenotypes; modulate inter-bacterial and host-microbiome interactions. The agent should achieve immunomodulatory action by supporting the host’s defense mechanisms, as well as regenerative effects, by the enhancement of wound healing and tissue regeneration.

Currently, collagen-based wound dressings are the most investigated solutions to obtain efficient coatings and to facilitate healing for both acute and chronic wounds. They can be in various forms, from hydrogels to solid dressings, and are applied directly on most wounds. The main advantages of such dressings rely on the fact that they facilitate wound repair by maintaining a suitable local environment. Numerous pharmaceutical companies are currently producing collagen and other polymeric dressings that contain various nanoparticles with antimicrobial effect and that are considered to support wound healing by avoiding microbial colonization and biofilm formation [61,62]. Such nanosystems embedded into the bioactive coatings may trigger intrinsic antimicrobial effects (i.e., silver nanoparticles) or are represented by nanosized shuttles able to deliver and specifically release antimicrobial agents (including antibiotics, plant-derived antimicrobial compounds, natural and synthetic virulence modulators, and biofilm inhibitory agents) [63]. The schematic representation of an ideal bioactive wound dressing is given in Figure 2.

## 5. Nano-Solutions for Wound Management

Nanotechnology offers unprecedented opportunities and cutting-edge solutions to designing efficient biomedical approaches. Numerous nanomaterials have been developed for application in wound care and healing. Nanostructures are revolutionary compounds that aim to enhance the therapeutic delivery of growth factors, antimicrobial agents, gene therapy vectors, and others to the wound [7].

**Figure 3:**
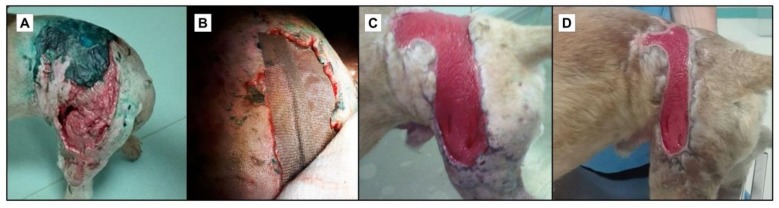
Clinical application of silver-containing impregnated dressing for wound healing. (**A**) Initial stage in which the wound is highly infected, a stage at which Dr. Laurentiu Leica took into consideration the possibility of skin grafting; (**B**) The use of a silver-containing impregnated polyamide dressing; (**C**,**D**) The beneficial effect of the dressing at 2 weeks and at one month, respectively, of follow-up. It was observed that the dressing not only exhibited antimicrobial effects but also promoted wound healing.

The most explored approaches for the management of wounds refer to the development of nano-devices for (i) inflammation control, (ii) cellular proliferation and re-epithelization, and (iii) tissue remodeling [64]. For all of the above-mentioned strategies, the proposed nanosystems include a variety of nanomaterials, starting with simple nanoparticles (inorganic and organic), functionalized nanoparticles, bioactive fibrous nanosystems (nanoscaffolds), and sphere-like nanoparticle embedding systems. Moreover, in chronic wounds, the main solutions refer to the optimization of nanomaterials able to avoid chronic microbial contamination of the wound and to also inhibit/disrupt the biofilms that usually form at the site of chronic wounds and delay or reduce significantly the healing chances (Figure 3). Recent advances made in the development of nanostructured wound coatings are presented in Table 2.

## 6. Antimicrobial Nanoparticles

Nanoparticles have been widely used as promising candidates for wound treatment. In the last years, nanotechnology has gained a lot of attention in the biomedical field. As an example, metallic silver nanoparticles have been used as antimicrobial agents against pathogenic bacteria that have exhibited resistance to multiple antibiotics [73]. As a result, nanotechnology has several advantages in the treatment of bacterial infections. Nanoparticles can be used in different forms to fight bacterial infections, such as coatings on implantable devices or biomaterials that not only have antibacterial properties but also enhance wound healing, antibiotic delivery systems, microbial diagnosis through bacterial detection systems, and vaccines. Even though the antimicrobial mechanisms of nanoparticles are not fully understood, there are several accepted processes, which include induction of oxidative stress, metal ion release, and non-oxidative means. It has been observed that these three mechanisms occur at the same time. Studies have suggested that silver nanoparticles (AgNPs) effectively neutralize the electric charge on the surface of the bacterial membranes, which disrupts its permeability, therefore resulting in bacterial death. Furthermore, the cell membrane can also be affected by the generation of reactive oxygen species (ROS) that suppress the antioxidant defense system [74].

Bacterial membrane damage is attained by electrostatic binding of the NPs with the bacterial cell wall. This process damages the integrity, potential, and, therefore, the depolarization of the membrane, resulting in the loss of the primary functions of the cell such as respiration, lysis, and energy conversion, which ultimately leads to cell death. It is considered that the production of ROS is the most effective way to induce cytotoxicity by NPs; it can be obtained either indirectly by a perturbation in the respiratory chain or directly by the NPs. High concentrations of ROS cause cell death and can even damage DNA or induce mutations [75].

### 6.1. Inorganic Nanoparticles

Inorganic metallic and oxide nanoparticles have numerous applications in the biomedical and pharmaceutical industries. Certain types, such as silver (Ag), copper (Cu), titanium, (Ti), iron (Fe), and zinc oxide (ZnO), have shown significant antimicrobial activity [76]. In addition, such nanoparticles have a wide antimicrobial spectrum and do not interfere with the selection of resistant mutants, being efficient also against biofilms and antibiotic-resistant isolates [77].

Silver nanoparticles (AgNPs) have been known for decades to have strong bactericidal effects, broad-spectrum antimicrobial activity, and, importantly, they are currently utilized for the therapy of both acute and chronic wounds in various preparations.

Silver-based formulations are utilized for avoiding microbial contamination and for the treatment of chronic wounds, such as ulcers and burns (Figure 3). Long ago antibiotics were introduced in antimicrobial therapy [78]. Currently, numerous wound coatings, which contain AgNPs, are available on the market for the treatment of wounds [79,80]. AgNPs represent an antimicrobial alternative for the future since they have numerous mechanisms of action against microbial cells. They are able to induce membrane pores and can also activate the production of reactive oxygen species (ROS) response within the cells, which leads to the death of the microorganisms [79,80]. AgNPs can release active ions at the wound site, which can penetrate the tissues but also the preformed biofilms, and can induce toxic effects in biofilm-embedded cells, thus destabilizing mature biofilms [81].

Along with their antimicrobial impact, AgNPs can promote wound healing via immune regulation. Innovative hybrid scaffolds made of metallic nanosilver particles–collagen/chitosan are able to regulate fibroblast migration and macrophage activation in a rat model. The healing mechanism seems to rely on the accelerated migration of fibroblasts and increased expression of α-smooth muscle actin (α-SMA) triggered by the nanostructured scaffold. Furthermore, in vivo studies showed increased levels of pro-inflammatory and scar-related factors, as well as α-SMA, while markers for macrophage activation were upregulated [82].

Such nanostructured coatings have the potential to fight against resistant wound infections, both in chronic and acute patients.

Zinc oxide (ZnO) nanoparticles have also been proven to enhance antimicrobial properties, being efficient in vitro, and to treat wound infections in vivo [83]. The main antimicrobial mechanism of ZnO nanoparticles is correlated with the production and release of ROS. Depending on their size, such nanoparticles may enter the bacteria cells and activate ROS production, which determines protein and DNA damage and thus cell death. Despite all these findings, the exact antimicrobial mechanism of ZnO nanoparticles is yet to be elucidated [84]. Moreover, recent studies have proven that ZnO nanoparticles may inhibit biofilm formation of relevant pathogenic and opportunistic bacteria, such as *P. aeruginosa*. Biofilm inhibition mechanism is still unknown for these nanoparticles but studies have revealed that they also impair the production of other virulence factors, such as pigments and quorum sensing (QS) molecules [85].

ZnO nanoparticles were proven to be efficient in preventing wound infection in mice and they are currently being intensively investigated for antimicrobial wound dressings [83]. Zinc is documented to possess great benefits for skin regeneration and ZnO nanoparticles were proven to enhance wound healing by possessing an anti-inflammatory effect, along with its antimicrobial impact [86]. Current trends in wound management are to develop biocompatible polymeric coatings and dressings containing ZnO nanoparticles, with no cytotoxic effects, that promote healing and inhibit microbial colonization at the wound site.

Although Ag and ZnO nanoparticles are the most investigated for advanced wound dressings and coatings, recent trends are considering other nanoparticles in the design of polymeric matrices for promoting wound healing of particular types of wounds. Magnetite, silica, and copper nanoparticles are being intensively investigated for their great biomedical potential and antimicrobial properties.

Magnetic nanoparticles (MNPs) have been widely studied and used in biomedical applications due to their distinctive properties, among which the most important being their ability to be controlled using an external magnetic field and the minor residual magnetism when the magnetic field is removed. Magnetic vehicles in the nanometer range have gained attention because of their high specific area and low internal diffusion resistance. In terms of chronic wound healing applications, Fe_3_O_4_ NPs loaded onto bacterial cellulose scaffolds have proven to be a promising candidate. Bacterial cellulose (BC) promotes wound healing through the gathering of extracellular matrix that accelerates contraction. When adding human adipose-derived stem cells (hASCs) into this matrix, it was observed that the magnetite–BC-based nanostructure promotes cellular proliferation, which is a key property for wound healing applications [87].

Fe_3_O_4_ and gold (Au) NPs have been intensively researched as antimicrobial nanomaterials. Separately, these nanoparticles are considered inert and do not possess antimicrobial features. However, these materials when reduced to nanometer size in particle form can be altered to exhibit antimicrobial properties. Microbiological studies on Fe_3_O_4_ have shown that modifying surfaces with these NPs have proven to have anti-adherent properties and considerably lower bacterial colonization for both Gram-negative and Gram-positive bacteria. It has been reported that when functionalized via photothermal process, AuNPs and nanorods exhibit antibacterial properties. When compared with Ag, AuNPs exhibit very low or even no antimicrobial effects on their own. However, Au nanomaterials can bind to several antibiotics, such as ampicillin, or even other nanomaterials. Combining amino-substituted pyrimidines and citrates with Au nanomaterials, together with light energy leads to ROS production, which is an effective antimicrobial mechanism. One of the reasons why Au has been intensively researched is its stability compared to other metallic NPs [75].

Silica nanoparticles (SiO_2_ NPs) have shown antibacterial properties, exhibiting a very high rate (99.9%) against *P. aeruginosa* and *E. coli*. Moreover, it was shown that their fibroblast proliferation inhibition rate is significantly lower than conventionally used antiseptics with proven wound healing benefits. Nitric oxide-releasing silica nanoparticles have been investigated in regards to wound healing and have shown that they play an important role in the deposition of extracellular matrix (ECM) proteins and cell proliferation [88]. Silica NPs have also been investigated as a gene delivery system for the treatment of cutaneous chronic wounds. Nanocomposites consisting of silica and collagen released interleukin 10 (IL-10) proving to be promising for the treatment of local cutaneous chronic wounds due to the high cellular uptake, cell targeting, and DNA protection [89].

Copper nanoparticles have attracted attention for wound healing applications due to their ability to promote angiogenesis [90]. However, an increase in copper salts or oxides to the wound bed has raised toxicity concerns. Therefore, several studies have investigated stabilizing the copper NPs. The incorporation of folic acid was proven to reduce cytotoxicity and promote cell migration. The system enhanced angiogenesis, induced collagen deposition, and, most importantly, increased wound closure rates [91].

Magnesium oxide (MgO) NPs demonstrated antimicrobial behavior against Gram-negative and Gram-positive bacteria, spores, and viruses. Apart from ROS induction, nanomaterials containing Mg have a direct effect on the inhibition of vital bacterial enzymes, for example, MgF_2_ effectively inhibits the biofilm formation of *Escherichia coli* and *Staphylococcus aureus*. Moreover, MgO NPs have the advantage of being cost effective [92].

Nitric oxide (NO) NPs are directly involved in several antimicrobial mechanisms. Their antibacterial properties are directly related to their shape and size. Even though NO NPs exhibit several advantages, their clinical applicability is restricted because they are highly reactive. However, they can still be used for their antibacterial properties when encapsulated, especially in controlled delivery systems [93].

### 6.2. Organic Nanoparticles

Organic NPs have several antimicrobial properties including releasing antibiotic and antimicrobial agents or penetrating cell membranes using cationic groups. The latter requires certain specifications regarding the length of the hydrophobic chain in order to effectively penetrate the bacterial membrane. However, the antimicrobial effect can also be attained by a highly positive surface charge, irrespective of the chain length. At high temperatures, organic materials have exhibited less stability compared to inorganic materials. This can lead to manufacturing issues especially when the required material needs to be stable and tolerate severe manufacturing conditions. Thus, inorganic materials in the nanometer scale are generally used in antimicrobial applications [75].

Polymeric antibacterial agents in the nanometer range are known for their long-lasting antimicrobial activity. Some of their advantages include chemical stability, non-volatility, and non-toxicity to biological membranes, e.g., skin. Polycationic antimicrobial agents exhibit antimicrobial activity due to their active groups and high surface density. Quaternary ammonium compounds have been reported to effectively act against Gram-positive and Gram-negative bacteria. For example, quaternary ammonium polyethylenimine (QPEI) NPs, when integrated into different polymeric matrices, display antimicrobial activity against several bacterial targets. Interestingly, QPEI NPs have the ability to induce cell death through an intracellular death signal. Even though this signal has not been identified yet, it determines cell death in layers of the biofilm that are not even in direct connection with the NPs [75].

Among several organic NPs, chitosan NPs have exhibited antimicrobial, antiviral, and antifungal action. The advantages of using chitosan include its biocompatibility, antimicrobial features, and low immunogenicity. The antimicrobial activity of chitosan NPs depends on various factors such as pH and solvent. It has been reported that it inhibits the activity of metallic NPs; therefore, it is used mainly with antibiotics. The antimicrobial activity of chitosan NPs is not yet fully understood [94]. 

## 7. Conclusions

With the increasing incidence of chronic biofilm infections, characterized by tolerance and resistance to antimicrobials, there is an imperative need for alternative therapeutic agents. An ideal approach to defeating polymicrobial wound infections should be based on a better understanding of the interbacterial and host–microbiome complex interactions, aiming to disrupt the pathogenic mechanisms and to enhance a beneficial environment. This approach should combine multiple complementary therapies with antimicrobial, immunomodulatory, and regenerative effects.

Innovative approaches in the areas of nanomedicine and nanotechnology could change the future of chronic wound management. Next generation wound dressings include nanocoatings that contain nanoparticles with intrinsic antimicrobial properties, active at low concentrations toward a large variety of infectious agents, and that are unlikely to provoke the emergence of resistance. Moreover, nanoparticles can act as drug carriers for other antimicrobial agents (such as plant-derived compounds, bacteriophages, antimicrobial peptides), microbiome regulators (probiotics, prebiotics), or agents that could accelerate wound healing (i.e., growth factors, stem cells etc.).

The coming years will be critical for the development of novel therapies and strategies for wound healing. Now there are opportunities to explore novel approaches and design new coating systems that will lead to further discoveries.

## Figures and Tables

**Table 1:** Wound therapy approaches.

Therapeutic Approach	Advantages	Disadvantages	Indication	Examples
**Wound debridement**	- disruption of the mucopolysaccharide matrix - destabilizes the biofilm’s architecture - promotes bacterial detachment - increases antimicrobial delivery	- may promote the inoculation of infection in deeper tissues	- chronic wounds	- mechanical - enzymatic - biological
**Topical or systemic antibiotic therapy**	- low cost - efficient in acute infections - eradication of planktonic bacteria - biofilm disruption	- development of antibiotic resistance - development of antibiotic tolerance in biofilms - microbial imbalance (target both pathogenic and beneficial bacteria) - multiple side effects	- acute wounds	- fluoroquinolones, tetracycline, rifampin, daptomycin, and vancomycin
**Topical antiseptic therapy**	- cytotoxic toward bacteria, fungi, and other microorganisms	- might damage host cells and adversely affect wound healing	- acute wounds - chronic wounds	- povidone-iodine, chlorhexidine, hydrogen peroxide, boric acid, silver sulfadiazine or nitrate, sodium hypochlorite, mafenide acetate, octenidine dihydrochloride, polyhexamethylene biguanide (polyhexanide)
**Bacteriophage therapy**	- maybe efficient against polymicrobial biofilm-mediated infections - very specific for targeted bacterial species	- biological-associated risks - unknown side effects - high production costs - applied in low amount as a topic treatment	- chronic wounds	- monophage preparations (staphylococcal bacteriophages, pyocianic bacteriophages) - multiple phage preparations
**Antimicrobial peptides**	- antimicrobial properties	- used in small amounts - low stability - increased volatility	- chronic wounds	- defensins, magainins, cecropins
**Probiotic therapy**	- accelerates wound healing - prevents wound colonization, biofilm development - interferes with the quorum sensing of *P. aeruginosa*	- insufficient data	- chronic wounds	- Lactobacillus plantarum

**Table 2:** Nanostructured coatings for wounds.

Type	Nanostructure(s)	Application	Mode of Action	Reference
Bioactive wound coating	Magnetite (Fe_3_O_4_) nanoparticles (NPs) and patchouli essential oil	Acute and chronic wound dressing	Inhibition of microbial colonization and biofilm formation	[65]
Nanophyto-modified wound dressing	Nanofluid-based Fe_3_O_4_ doped with eugenol and limonene	Fixed layer on a regular external wound cover	Anti-adherence and anti-biofilm properties against bacterial pathogens	[66]
Layer-by-layer (LBL) electrostatic self-assembled antimicrobial nanocoating	Chemically modified cotton substrate and copper-based NP layer	Metal-based wound care and inhibition of pathogenic bacterial infections	Inhibition of *A. baumannii* (multidrug resistant bacterial wound pathogen)	[67]
Bioactive wound coating	Silver NPs for polyester–nylon wound dressing	Reduction of exogenous microbial colonization of wound dressing	Inhibition of microbial colonization, attachment, and biofilm growth	[68]
Bioactive wound coating	Nano bacterial cellulose and sesame oil	Modern wound dressing	Improved healing properties and inhibition of bacterial infections	[69]
Nano-coated wound dressing	Fe_3_O_4_ and *Satureja hortensis* (SO) essential oil	Cutaneous wound dressing	Inhibition of fungal biofilm development and adherence of *C. albicans*	[70]
Nano-coated wound dressing	Silver nanocoating on cotton gauzes	Acute and chronic wound dressing	Reduction of bacterial growth and biofilm proliferation	[71]
Bioactive wound coating	Nano-silver-coated microfibrous eggshell membrane	Cutaneous wound dressing	Antibacterial and anti-inflammatory activity, and also acceleration of wound healing	[72]
